# Research on Teleconsultation service quality based on multi-granularity linguistic information: the perspective of regional doctors

**DOI:** 10.1186/s12911-020-01155-5

**Published:** 2020-06-18

**Authors:** Wei Lu, Xin-pu Wang, Jie Zhao, Yun-kai Zhai

**Affiliations:** 1 School of Management Engineering, Zhengzhou University, No.100 Science Avenue, Zhengzhou, 450001 China; 2 The First Affiliated Hospital of Zhengzhou University, Zhengzhou, 450052 China; 3 National Engineering Laboratory for Internet Medical Systems and Applications, Zhengzhou, 450052 China

**Keywords:** Teleconsultation, Service quality assessment, Multi-granularity linguistic information, Regional doctors

## Abstract

**Background:**

Due to the increasing complexity in socioeconomic environments and the ambiguity in human cognition, decision makers prefer to give linguistic cognitive information with different granularities according to their own preferences. Consequently, to consider the uncertainty and preferences in the evaluation process, a method based on Multi-Granularity Linguistic Information (MGLI) for evaluating teleconsultation service quality is proposed, which provides a new research direction for scientific evaluation and improvement of teleconsultation service quality.

**Methods:**

Firstly, this paper explored a service quality evaluation system from the perspective of regional doctors. And then considering the uncertainty and preferences of decision makers, MGLI was used to optimize the index system according to the similarity degree between the linguistic evaluation information and a given linguistic term set. Finally, the empirical research was conducted using Henan Province Telemedicine Center of China (HTCC) as an example to identify the direction for improving the service quality in teleconsultation.

**Results:**

This study found that the number of consulting rooms, attitude of operators, consultation duration, charges, and attitude of experts are the key factors affecting the quality of teleconsultation service.

**Conclusions:**

Suggestions for improving the quality of teleconsultation service are put forward in terms of optimizing the allocation of consulting rooms, improving regional doctors’ experience and standardizing charging standards, which provides a new direction for improving the quality of teleconsultation service.

## Background

With the increasing demand for medical care, limited medical resources inevitably lead to problems such as inadequate and overly expensive medical services [1]. However, the emergence of telemedicine has solved the problem of uneven distribution of medical resources to a certain extent. Teleconsultation, the main form of telemedicine, uses information technology to achieve long-distance clinical health care [2], which promotes the sinking of high-quality medical resources and improves the overall efficiency of the health care system [3]. The rapid development of teleconsultation is bound to bring about the demand for quality control, and quality of service is the critical factor to attract public and healthcare consumers. Therefore, identifying the influencing factors of service quality in teleconsultation, conducting scientific evaluation and improving its service quality have become a widespread concern for government departments, medical and health institutions, doctors, patients and so on.

The existing research achievements related to the service quality of teleconsultation are abundant. This study used the “Web of Science Core Collection” database as the international literature source to conduct a bibliometric analysis on the research of teleconsultation. We chose “teleconsultation” or “‘remote consultation’” or “teleconsulting” or “telemedicine” or “telehealth” and “service evaluation” as the keywords for literature retrieval. The literature type was selected as “Article” and the retrieval time was May 4, 2020. A total of 10,380 search results were obtained. The results show that service quality of teleconsultation in existing studies have been generally divided into four major groups: practical effect evaluation (clinical outcomes and implementation effects), satisfaction statistics, economy and feasibility verification, and theoretical studies. Randomized trials were used to evaluate the feasibility of teleconsultation and its clinical value in rural areas [4–6]. Diniz et al. [7] and von Wangenheim et al. [8] calculated the satisfaction rate of teleconsultation. Seto et al. [9] conducted an evaluation of the telehealth system for nurses and patients who had used telehealth to determine the direction of future improvements through a mixed method. Teleconsultation avoids unnecessary referrals and misdiagnosis, resulting in significant potential for cost savings and improvement of patient experience [10]. Some existing studies have assessed the economic benefits and feasibility of telehealth through comparative analysis [11–13]. Zennaro et al. [14] evaluated the effect of teleconsultation on the number of in-hospital consultations for fracture patients in a prospective study and confirmed that teleconsultation can reduce the nursing cost of healthcare system. Rasmussen et al. [15] verified the effectiveness of teleconsultation through the comparative analysis of the effect of teleconsultation and conventional clinic treatment for diabetic patients, and found that teleconsultation is a safe and feasible option. In addition, the theoretical studies on conceptual framework for telehealth program evaluation were also concerned [16, 17].

As aforementioned, in terms of research perspectives, scholars have focused on the quality of teleconsultation service from the perspective of patients, however, regional doctors are the main participants in teleconsultation, a literature gap exists in evaluating the quality of teleconsultation service from the perspective of real users (regional doctors). For influencing factors, teleconsultation provides long-distance traditional medical service with modern communication technologies, and existing studies have proved that the quality of teleconsultation process is also an important factor that cannot be ignored [18], while only a very limited number of studies incorporate the impact of mobile platform quality and the process quality. As for evaluation methods, scholars often use comparative experiments or give evaluation information with exact values to evaluate the service quality [19]. In reality, however, due to the uncertainty and ambiguity of human cognition and the complexity of the service, decision makers prefer to choose different linguistic evaluation term sets with different cardinalities (Multi-Granularity Linguistic Information, MGLI) according to their own preferences, rather than give exact evaluation values. For instance, in practical problems, some experts are willing to use a linguistic term set with five terms (e.g., S_0_: fail, S_1_: pass, S_2_: good, S_3_: very good, S_4_: excellent), while others prefer to use the one with seven terms (e.g., S_0_: very poor, S_1_: poor, S_2_: slight poor, S_3_: fair, S_4_: slight good, S_5_: good, S_6_: very good). It should be noted that a small-granularity linguistic term set is beneficial for the experts to express clear evaluation information, while a large-granularity linguistic term set can provide experts with more choices to express their accurate assessment information [20]. Consequently, the research on evaluation problem with MGLI is very important to practical applications, which makes up the shortcomings of the existing studies that only taking the exact evaluation information into consideration. The evaluation of linguistic information has been effectively verified in other service domains such as e-commerce logistics [21], advanced teaching [22], mobile services [23]. As previously noted, the MGLI, which can provide more decision information for decision makers, is introduced and adopted to optimize the index system and evaluate the service quality of teleconsultation in this paper.

Accordingly, a method based on MGLI for evaluating teleconsultation service quality from the perspective of regional doctors is proposed in this paper. Firstly, a multidimensional evaluation index system for teleconsultation service quality is constructed from the perspective of regional doctors. And then we introduce MGLI to optimize the indicator system based on the similarity degree between the linguistic evaluation information and a given linguistic term set. An optimized evaluation index system is developed. Finally, taking the “5GAP Model” as the core, the empirical research is conducted by linguistic information to identify the direction for improving the quality of teleconsultation service, thereby specific suggestions are proposed in a targeted manner, which provides a reference for improving the service quality in teleconsultation.

## Methods

Let *C* = {*C*_1_, *C*_2_, …, *C*_*l*_} be a user set, where *C*_*k*_ is the *k-th* user, *k* = 1, 2, …, *l*. Let *I* = [*I*_*ij*_]_*m* × *n*_ be a matrix of service quality indicators, where *I*_*ij*_ is the *j-th* indicator in the *i-th* dimension, *i* = 1, 2, …, *m*; *j* = 1, 2, …, *n*. Let $P={\left[{P}_{ij}^k\right]}_{m\times n}$ and $E={\left[{E}_{ij}^k\right]}_{m\times n}$ be perception matrix and expectation matrix respectively, where ${P}_{ij}^k$ (${E}_{ij}^k$) is the *k-th* user’s perception (expectation) of the *j-th* indicator in the *i-th* dimension.

### Linguistic information

It is difficult for decision makers to evaluate the service quality with exact numerical values because of the uncertainty and ambiguity of thinking, therefore, they prefer to use the linguistic information rather than give the numerical values for evaluation. Let S = {S_t_}, t ∈ {0, 1, 2, 3, …, T} be a linguistic evaluation information set, *S*_*t*_ is defined as the *t-th* evaluation information. It is usually required that the linguistic term set satisfies the following additional characteristics.

(1) It is ordered: *S*_*i*_ ≥ *S*_*j*_(*i* ≥ *j*);

(2) There is a negative operator: *Neg*(*S*_*i*_) = *S*_*j*_, *j* = *T* − *i*;

(3) There is a maximize operator: if *S*_*i*_ ≥ *S*_*j*_, then *Max*(*S*_*i*_, *S*_*j*_) = *S*_*i*_;

(4) There is a minimize operator: if *S*_i_ ≤ *S*_*j*_, then *Min*(*S*_*i*_, *S*_*j*_) = *S*_*i*._

The number of *S* determines the granularity of the set. If *S* = 7, the linguistic information is a set with seven terms, which is specifically expressed as

$$ {\displaystyle \begin{array}{l}S=\left\{{\mathrm{S}}_0,{\mathrm{S}}_1,{\mathrm{S}}_2,{\mathrm{S}}_3,{\mathrm{S}}_4,{\mathrm{S}}_5,{\mathrm{S}}_6\right\}\\ {}=\Big\{\mathrm{very}\ \mathrm{poor}/\mathrm{very}\ \mathrm{unimportant},\mathrm{poor}/\mathrm{unimportant},\mathrm{slight}\mathrm{ly}\ \mathrm{poor}/\mathrm{slight}\ \mathrm{unimportant},\\ {}\mathrm{fair}/\mathrm{middle},\mathrm{slight}\ \mathrm{good}/\mathrm{slight}\ \mathrm{important},\mathrm{good}/\mathrm{important},\mathrm{very}\ \mathrm{good}/\mathrm{very}\ \mathrm{important}\Big\}\end{array}} $$

Decision makers usually give evaluation information according to the pre-established linguistic term set, but sometimes evaluation information may exceed the scale of a given set. Hence an extended linguistic evaluation set $\overline{S}=\left\{{\mathrm{S}}_{\mathrm{t}}|0\le \mathrm{t}\le \mathrm{T}\hbox{'}\right\}$ is defined to avoid data loss, and *S*_0_, *S*_*T*_ are the upper and lower limit of *S*_*t*_ respectively. If S_t_ ∈ *S*, the evaluation term is in the original linguistic evaluation set. If ${\mathrm{S}}_t\in \overline{S}$, the evaluation information is in the extended linguistic evaluation set.

After collecting the linguistic evaluation information, the information needs to be converted into corresponding fuzzy numbers for data analysis and calculation. Triangular fuzzy numbers and trapezoidal fuzzy numbers are the most common fuzzy numbers. The main difference is that the former is represented by an exact value, and the latter is described by an interval, which is sufficient to capture the uncertainty information of the language. Therefore, to obtain more comprehensive linguistic evaluation information of teleconsultation, this paper uses trapezoidal fuzzy numbers to accomplish the conversion of linguistic information to numerical information.

### Trapezoidal fuzzy numbers

Let *R* be a set of real numbers. If *A* = (*a*, *b*, *c*, *d*); *a*, *b*, *c*, *d* ∈ *R*, ‐ ∞  < a ≤ *b* ≤ *c* ≤ *d* <  + ∞, then *A* is a trapezoidal fuzzy function, where *a* is the lower limit and *d* is the upper limit. If *a* > 0, *A* is a positive trapezoidal fuzzy function. If *b* = *c*, it degenerates into triangular fuzzy numbers. And if *a* = *b*, *c* = *d*, the *A* deteriorates into general fuzzy interval numbers. The membership function of the trapezoidal fuzzy function *μ*_*A*_ : *R* → [0, 1] satisfies the following conditions.

1 $$ {\mu}_A(x)=\left\{\begin{array}{c}\frac{x-a}{b-a},a\le x<\mathrm{b}\\ {}1,b\le x\le c\\ {}\frac{x-d}{c-d},c<x\le b\\ {}0, other\end{array}\right. $$

Where *μ*_*A*_(*x*) represents the qualification that element *x* belongs to the fuzzy subset A. The value of *μ*_*A*_(*x*) ranges from 0 to 1, indicating that the qualification is from small to large. If *μ*_*A*_(*x*) = 0 or *μ*_*A*_(*x*) = 1, the fuzzy set degenerates into a classical set. There are two positive trapezoidal fuzzy numbers *A*_1_ = (*a*_1_, *b*_1_, *c*_1_, *d*_1_) and *A*_2_ = (*a*_2_, *b*_2_, *c*_2_, *d*_2_), which follow the following algorithms.

2 $$ {A}_1\pm {A}_2=\left({a}_1,{b}_1,{c}_1,{d}_1\right)\pm \left({a}_2,{b}_2,{c}_2,{d}_2\right)=\left({a}_1\pm {a}_2,{b}_1\pm {b}_2,{c}_1\pm {c}_2,{d}_1\pm {d}_2\right) $$

3 $$ {A}_1\times {A}_2=\left({a}_1,{b}_1,{c}_1,{d}_1\right)\times \left({a}_2,{b}_2,{c}_2,{d}_2\right)=\left({a}_1{a}_2,{b}_1{b}_2,{c}_1{c}_2,{d}_1{d}_2\right) $$

4 $$ {A}_1\div {A}_2=\left({a}_1,{b}_1,{c}_1,{d}_1\right)\div \left({a}_2,{b}_2,{c}_2,{d}_2\right)=\left({a}_1/{a}_2,{b}_1/{b}_2,{c}_1/{c}_2,{d}_1/{d}_2\right) $$

5 $$ \lambda {A}_1=\lambda \left({a}_1,{b}_1,{c}_1,{d}_1\right)=\left(\lambda {a}_1,\lambda {b}_1,\lambda {c}_1,\lambda {d}_1\right)\left(\lambda \ge 0\right) $$

For uncertain linguistic information *S* = [*S*_*m*_, *S*_*n*_], the method of converting trapezoidal fuzzy numbers is as follows.

6 $$ A=\left({a}_1,{a}_2,{a}_3,{a}_4\right)=\left(\max \left\{\frac{2m-1}{2T+1},0\right\},\frac{2m}{2T+1},\frac{2n+1}{2T+1},\min \left\{\frac{2n+2}{2T+1},1\right\}\right) $$

Some scholars have presented that segmentation integral method can be used to realize the defuzzify of trapezoidal fuzzy numbers [24], which is defined as

7 $$ {\displaystyle \begin{array}{l}P(A)={\int}_0^1\frac{y\left({L}^{-1}(y)+{R}^{-1}(y)\right)}{2} dy/{\int}_0^1 ydy\\ {}={\int}_0^1\frac{y\left({a}_1+\left({a}_2-{a}_1\right)y+{a}_4+\left({a}_3-{a}_4\right)y\right)}{2} dy/{\int}_0^1 ydy\\ {}=\frac{1}{6}\left({a}_1+2{a}_2+2{a}_3+{a}_4\right)\end{array}} $$

### Index system optimization

MGLI is used to optimize the initial evaluation index system. Firstly, experts choose linguistic evaluation term sets with different cardinalities (MGLI) according to their own preferences to reveal their clear assessment information. Secondly, the linguistic information and uncertain linguistic interval are converted into corresponding trapezoidal fuzzy numbers according to Eq.(6). Let *W* = (*ω*_1_, *ω*_2_, …, *ω*_*f*_)^*T*^ be the weight vector of experts, $V={\left[{V}_{ij}^k\right]}_{m\times n}$ be a matrix of evaluation values, where ${V}_{ij}^k$ is the importance score of the *k-th* expert for the *j-th* indicator in the *i-th* dimension. Then the average evaluation value of each indicator is calculated.

8 $$ {\displaystyle \begin{array}{l}\overline{V_{ij}^k}=\left(\sum \limits_{k=1}^f{\omega}_k\times {V}_{ij}^k\right)/\sum \limits_{k=1}^f{\omega}_k\\ {}=\left(\left[{S}_{p1},{S}_{q1}\right]\times \left[{S}_{m1},{S}_{n1}\right]+\dots +\left[{S}_{pf},{S}_{qf}\right]\times \left[{S}_{mm},{S}_{nm}\right]\right)/\left(\left[{S}_{p1},{S}_{q1}\right]+\dots +\left[{S}_{pf},{S}_{qf}\right]\right)\end{array}} $$

Subsequently, the evaluation index system is optimized according to the similarity degree between the linguistic evaluation information and a given linguistic evaluation term set. Let ${M}_{\theta }=\left({m}_{\theta}^1,{m}_{\theta}^2,{m}_{\theta}^3,{m}_{\theta}^4\right),\theta =0,1,2,\dots, g$ be the trapezoidal fuzzy numbers corresponding to a linguistic evaluation term set. The equation of similarity degree is as follows.

9 $$ Y\left({f}^{\ast },{M}_{\theta}\right)=1-\frac{1}{4}\sum \limits_{h=1}^4\left|{f}^{\ast }-{M}_{\theta}^h\right| $$

Finally, the evaluation index system is optimized. Remaining the indicators with the highest similarity degree to the “slight important”, “important” and “very important”. Removing the indicators with the highest similarity degree to the “slight unimportant”, “unimportant” and “very unimportant”. Combining the rationality of indicators and expert opinions, the indicators with the highest similarity degree to “middle” are appropriately integrated to optimize the index system.

### Service quality evaluation

The “5GAP Model” is a tool for analyzing the root cause of service quality gaps, indicating that the gaps between perception and expectation of customers can identify the direction of service quality improvement. Therefore, the gaps between perception and expectation of customers are used to identify shortcomings in service quality in this paper.

Converting the collected linguistic evaluation information into trapezoidal fuzzy numbers according to Eq. (6). Then sorting out the data based on Eqs. (1)–(5), and calculating the average perceptual evaluation values, average expected evaluation values and their gaps(Eqs. (10)–(11)).

10 $$ {\displaystyle \begin{array}{l}\overline{P_{ij}}=\left(\sum \limits_{k=1}^l{P}_{ij}^k\right)/l=\left(\left[{S}_{m1},{S}_{n1}\right]+\dots +\left[{S}_{mm},{S}_{mn}\right]\right)/l=\left({S}_{m1}+\dots +{S}_{mm},{S}_{n1}+\dots +{S}_{nm}\right)/l\\ {}=\left[\left({S}_{m1}+\dots +{S}_{mm}\right)/l,\left({S}_{n1}+\dots +{S}_{nm}\right)/l\right]\end{array}} $$

11 $$ \overline{E_{ij}}=\left(\sum \limits_{k=1}^l{E}_{ij}^k\right)/l=\left[\left({S}_{a1}+\dots +{S}_{am}\right)/l,\left({S}_{b1}+\dots +{S}_{bm}\right)/l\right] $$

12 $$ GAP=\overline{P_{ij}}-\overline{E_{ij}} $$

In order to facilitate the analysis, the trapezoidal fuzzy numbers are converted into the exact values according to Eq.(7).

## Study design

MGLI is used to optimize the evaluation index system and evaluate the quality of teleconsultation service in this paper. The specific implementation processes are as follows.

**Step 1** According to literature research and the features of teleconsultation service, an initial multidimensional evaluation index system for teleconsultation service quality is developed.

**Step 2** MGLI is used to optimize the evaluation index system. Specifically, experts provide MGLI to evaluate the importance of indicators, and the multi-granularity uncertain linguistic evaluation information is converted into trapezoidal fuzzy numbers based on Eq. (6). Then calculating the similarity degree between linguistic evaluation information and a linguistic evaluation term set based on Eqs. (8)–(9) to identify the key indicators, and constructing a more optimized multidimensional evaluation index system.

**Step 3** Linguistic information is used to evaluate the service quality of teleconsultation. The empirical research is conducted using HTCC as an example in this section. The collected linguistic evaluation information is converted and sorted according to Eqs. (1)–(6), and the average perceptual values, average expected values and their gaps are calculated according to Eqs. (10)–(12). Then the Eq. (7) is used to accomplish the defuzzify, thereby identifying the direction for improving the service quality of teleconsulting and proposing suggestions to improve the service quality in a targeted manner.

## Results

### Initial index system construction

#### Research on dimensions

There are similarities between mobile service and teleconsultation service, and teleconsultation is a form of medical service. Therefore, the studies on mobile service and medical service quality provide important ideas and inspiration for the teleconsultation service evaluation. At present, the evaluation dimensions of mobile service quality have been extensively studied (in Table 1).

**Table 1:** Dimensions of service quality evaluation

Scholars	Evaluation dimensions
Al-Hubaishi [25]	System quality, Environmental quality, Information quality, Interaction quality, Network quality and Outcome quality
Lee et al. [26]	Satisfaction, Service quality, Risk, Willing to continue using
Wang et al. [27]	Interactive quality, Environmental quality, Outcome quality
Zhao and Guo [28]	Environment, Interaction, Control, Results
Kapoor and Vij [29]	Login time, Visual design, Navigational design, Information design, Collaboration and Service quality
Huang et al. [30]	Contact, Responsiveness, Fulfillment, Privacy and Efficiency

Nowadays, the evaluation of mobile service quality has been analyzed from multiple perspectives. Based on the Donabedian Assessment Theory of “structure-process-outcome” of traditional medical quality evaluation and the research of mobile service quality evaluation, the dimensions of teleconsultation service quality evaluation index system are initially determined as Network quality, System quality, Structure quality, Interaction quality, and Outcome quality.

#### Research on influencing factors

(1) Literature research.

Scholars have assessed the quality of service from various aspects. The European Health Organization Office has proposed a performance evaluation tool for hospital quality improvement, which includes four areas: clinical effectiveness, efficiency, the practicality of medical staff, and response management [31]. Clinical Indicator Program (CIP) [32] includes procedure-related, time-related, results-related, efficiency-related indicators and so on. There are four main types of indicators in the UK hospital rating system: medical services, clinical errors, patient satisfaction, and staff performances [33]. Japanese third-party assessment organizations assess the content of the hospital organizational structure, patient satisfaction, the quality of diagnosis and treatment, and the rationality of hospital management [34]. In addition, there is also a China Medical Care Quality Indicator System in China (CHQIS). This section summarizes the influencing factors of teleconsultation service quality into network quality, system quality, structure quality, interaction quality and outcome quality based on previous studies, as shown in Table 2.

**Table 2:** Initial evaluation index system of teleconsultation service quality

Dimensions	Operational definition	Sub-indicators	Reference sources
Network quality	Network speed and efficiency, reflecting service smoothness and user experience	Network Service Provider	[35, 36]
		Network Rate	[36]
System quality	Mobile platform performance and teleconsultation process quality	Video Resolution	[37]
		Equipment Quality	[38]
		Process Convenience	CIP [37, 39],
		Operational Ease of Use	CIP [37, 39],
Structure quality	Resource allocation and management of teleconsultation service	Doctor-Patient Ratio	CHQIS [32],
		Consultation Visitors	CHQIS [32],
		Turnover Rates of Consulting Room	CHQIS [32],
		Charges	[35, 37]
Interaction quality	The quality of interactions between regional doctors and experts, and between regional doctors and the platform	Purpose of Application	Teleconsultation feature
		Appointment Channel	Teleconsultation feature
		Waiting Time	[32, 38]
		Regional Hospital Level	[40]
		Data Integrity	Teleconsultation feature
		Regional Doctor Level	[33, 41]
		Expert Level	[39, 41]
		Operators’ Attitude	[35, 39, 41]
		Experts’ Attitude	[35, 39, 41]
		Consultation Duration	[42]
Outcome quality	The service effect actually perceived by the applicant	Information Usefulness	[39]
		Diagnostic Coincidence Rate	CIP [34, 38],
		Treatment Effect	CIP [34, 39],
		Re-Consultation Rate	[1]

(2) The features of teleconsultation.

Teleconsultation is a kind of medical activity carried out by service stations of medical institutions at all levels according to their business needs. The process includes the following three aspects.

##### Consultation application

Regional doctors apply for teleconsultation and upload medical records in the telemedicine information platform when there is demand from the regional medical institutions. Then the staff in the central hospital with better medical resources will contact and arrange experts to determine the consultation time. Due to the features of teleconsultation, the purposes of application are different, thereby regional doctors have different perception of service quality. The interaction channel is an important factor affecting the quality of interaction [39]. In addition, the response efficiency of the central hospital will directly affect the service quality in the first stage of teleconsultation. Therefore, waiting time, appointment channels and purpose of application will affect the quality of service during the consultation application period.

##### Formal consultation

These two parties will discuss and diagnose the patients’ medical records through video and audio equipment at the appointed time, obtaining diagnostic opinions and improving further treatment plans. In the process of communication between the two parties, the asymmetry of knowledge will hinder the communication [40], and the value of time, convenience and perceptual quality can positively affect the quality of service [42]. Therefore, we put the regional hospital level, doctors’ technical title, attitude and consultation time into the influencing factors of service quality.

##### Consultation evaluation

After the consultation, the service will be evaluated to reflect the quality of medical care and provide the theoretical basis for the development of teleconsultation. Information usefulness [39] and diagnostic coincidence rate [34, 38] are visual assessments of service quality and reflect the effectiveness of teleconsultation service. In addition, the treatment effect [34, 39] is the most direct reflection and evaluation of the service quality in teleconsultation. The re-consultation rate is an important indicator to measure the quality of medical service [1]. Thus, information usefulness, diagnostic coincidence rate, treatment effect and re-consultation rate will be included in the outcome category to measure the quality of teleconsultation service.

#### Initial evaluation indicators

This section constructs the initial evaluation index system according to the above literature analysis and characteristic analysis. The results are shown in Table 2.

### Index system optimization

In order to make the index system more optimized and reasonable, we invited experts, including regional doctors and health care professionals who had participated in teleconsultation activity, to evaluate the importance of each indicator. Then the indicators were reassessed, forming a more reasonable teleconsultation service quality evaluation index system based on the scoring results. Let *S* = {*S*_0_, *S*_1_, *S*_2_, *S*_3_, *S*_4_, *S*_5_, *S*_6_} be a 7-granularity linguistic term set, which was used to calculate the similarity degree with linguistic evaluation information of experts.

#### Evaluation information processing

First, the collected MGLI (see Supplementary Files) was converted into trapezoidal fuzzy numbers according to Eq. (6). The average evaluation values were obtained from Eq. (8). The experts selected for the survey have rich practical experience in teleconsultation, so we regarded the weight of each expert as equal. In this paper, the values of variables of Eq.(8) are *i* = 5, *j* = 24, *k* = 15.

Taking the “Network Service Provider” indicator as an example, the result is $\overline{V_{11}}=\frac{1}{15}\sum \limits_{k=1}^{15}{x}_{11}^k=\left(0.206,0.301,0.431,0.527\right)$. Similarly, the average trapezoidal fuzzy numbers of indicators are shown in Table 3.

**Table 3:** Average trapezoidal fuzzy numbers of indicators

Sub-Indicators	$\overline{x_{ij}}$	Sub-Indicators	$\overline{x_{ij}}$
Network Service Provider	(0.206,0301,0.431,0.527)	Waiting Time	(0.578,0.673,0.794,0.874)
Network Rate	(0.512,0.607,0.713,0.808)	Regional Hospital Level	(0.643,0.738,0.864.0.932)
Video Resolution	(0.608,0.703,0.823,0.896)	Data Integrity	(0.533,0.628,0.733,0.828)
Equipment Quality	(0.648,0.743,0.848,0.914)	Regional Doctor Level	(0.568,0.663,0.758,0.854)
Process Convenience	(0.412,0.507,0.602,0.697)	Expert Level	(0.810,0.905,1.000,1.000)
Operational Ease of Use	(0.392,0.487,0.582,0.677)	Operators’ Attitude	(0.568,0.663,0.758,0.854)
Doctor-Patient Ratio	(0.573,0.668,0.773,0.861)	Experts’ Attitude	(0.618,0.713,0.829,0.909)
Consultation Visitors	(0.432,0.528,0.623,0.718)	Consultation Duration	(0.533,0.628,0.723,0.811)
Turnover Rates of Consulting Room	(0.558,0.653,0.758,0.846)	Information Usefulness	(0.649,0.744,0.839,0.915)
Charges	(0.563,0.659,0.764,0.852)	Diagnostic Coincidence Rate	(0.668,0.764, 0.859,0.919)
Purpose of Application	(0.573,0.668,0.798,0.879)	Treatment Effect	(0.810,0.905, 1.000,1.000)
Appointment Channel	(0.226,0.321,0.462,0.557)	Re-consultation Rate	(0.427,0.522,0.617,0.712)

#### Similarity degree calculation

The corresponding trapezoidal fuzzy numbers set of the given 7-granularity evaluation term is *M*_*θ*_ = (*m*_*θ*_^1^, *m*_*θ*_^2^, *m*_*θ*_^3^, *m*_*θ*_^4^), *θ* = 0, 1, 2, 3, 4, 5, 6. Calculating the similarity degree between the linguistic evaluation information and the given evaluation term set according to Eq. (9). The results are shown in Table 4.

**Table 4:** The similarity degree

	*S*_0_	*S*_1_	*S*_2_	*S*_3_	*S*_4_	*S*_5_	*S*_6_
*Y*(*f*_1_^∗^, *M*_*θ*_)	0.6916	0.8264	**0.9641**	0.8661	0.7126	0.5586	0.4239
*Y*(*f*_2_^∗^, *M*_*θ*_)	0.3976	0.5324	0.6864	0.8399	**0.9768**	0.8526	0.7179
*Y*(*f*_3_^∗^, *M*_*θ*_)	0.3001	0.4348	0.5888	0.7423	0.8958	**0.9502**	0.8154
*Y*(*f*_4_^∗^, *M*_*θ*_)	0.2695	0.4042	0.5582	0.7117	0.8652	**0.9796**	0.8461
*Y*(*f*_5_^∗^, *M*_*θ*_)	0.5030	0.6378	0.7918	**0.9453**	0.9012	0.7472	0.6125
*Y*(*f*_6_^∗^, *M*_*θ*_)	0.5235	0.6582	0.8122	**0.9657**	0.8808	0.7268	0.5920
*Y*(*f*_7_^∗^, *M*_*θ*_)	0.3391	0.4739	0.6279	0.7814	**0.9349**	0.9112	0.7764
*Y*(*f*_8_^∗^, *M*_*θ*_)	0.4826	0.6173	0.7713	**0.9248**	0.9217	0.7677	0.6330
*Y*(*f*_9_^∗^, *M*_*θ*_)	0.3539	0.4887	0.6427	0.7962	**0.9497**	0.8964	0.7616
*Y*(*f*_10_^∗^, *M*_*θ*_)	0.3482	0.4830	0.6370	0.7905	**0.9440**	0.9020	0.7673
*Y*(*f*_11_^∗^, *M*_*θ*_)	0.3284	0.4631	0.6171	0.7706	**0.9241**	0.9219	0.7871
*Y*(*f*_12_^∗^, *M*_*θ*_)	0.6660	0.8007	**0.9523**	0.8918	0.7383	0.5843	0.4495
*Y*(*f*_13_^∗^, *M*_*θ*_)	0.3279	0.4626	0.6166	0.7701	**0.9236**	0.9224	0.7877
*Y*(*f*_14_^∗^, *M*_*θ*_)	0.2632	0.3979	0.5519	0.7054	0.8589	**0.9733**	0.8524
*Y*(*f*_15_^∗^, *M*_*θ*_)	0.3771	0.5119	0.6659	0.8194	**0.9703**	0.8731	0.7384
*Y*(*f*_16_^∗^, *M*_*θ*_)	0.3469	0.4817	0.6357	0.7892	**0.9427**	0.9033	0.7686
*Y*(*f*_17_^∗^, *M*_*θ*_)	0.1291	0.2639	0.4179	0.5714	0.7249	0.8789	**0.9864**
*Y*(*f*_18_^∗^, *M*_*θ*_)	0.3469	0.4817	0.6357	0.7892	**0.9427**	0.9033	0.7686
*Y*(*f*_19_^∗^, *M*_*θ*_)	0.2902	0.4250	0.5790	0.7325	0.8860	**0.9600**	0.8253
*Y*(*f*_20_^∗^, *M*_*θ*_)	0.3841	0.5189	0.6729	0.8264	**0.9773**	0.8661	0.7314
*Y*(*f*_21_^∗^, *M*_*θ*_)	0.2710	0.4057	0.5597	0.7132	0.8667	**0.9793**	0.8445
*Y*(*f*_22_^∗^, *M*_*θ*_)	0.2553	0.3901	0.5441	0.6976	0.8511	**0.9886**	0.8602
*Y*(*f*_23_^∗^, *M*_*θ*_)	0.1291	0.2639	0.4179	0.5714	0.7249	0.8789	**0.9864**
*Y*(*f*_24_^∗^, *M*_*θ*_)	0.4882	0.6229	0.7769	**0.9304**	0.9161	0.7621	0.6273

#### Key indicators identification

According to the Table 4, the evaluation value *f*_1_^∗^ and *S*_2_ have the highest similarity degree, *f*_2_^∗^ with the highest similarity degree to *S*_4_, and *f*_3_^∗^ has the highest similarity degree with *S*_5_. Then the importance of each indicator was identified, and the results were fed back to experts. Finally, the indicator system was further processed, mainly as follows.

(1) The indicators with the highest similarity degree to *S*_4_- *S*_6_ were remained. The indicators with the highest similarity degree to *S*_0_- *S*_2_ were removed, that is, “Network Service Provider” and “Appointment Channel” were removed.

(2) Taking into account the rationality of indicators and opinions of experts, the indicators with the highest similarity degree to *S*_3_ were optimized. We integrated “Process Convenience” and “Operational Ease of Use” into “Operational Convenience”. The combination of “Network Quality” and “System Quality” was collectively referred to as “System Quality”. It is an indicator to measure the system quality, such as network quality, platform quality, process quality. Both the “Doctor-Patient Ratio” and “Consultation Visits” reflect the allocation of medical resources, so we merged them into “Rationality of Doctor-Patient Ratio”. “Re-consultation Rate” was included in the “Treatment Effect”.

**Fig. 1:**
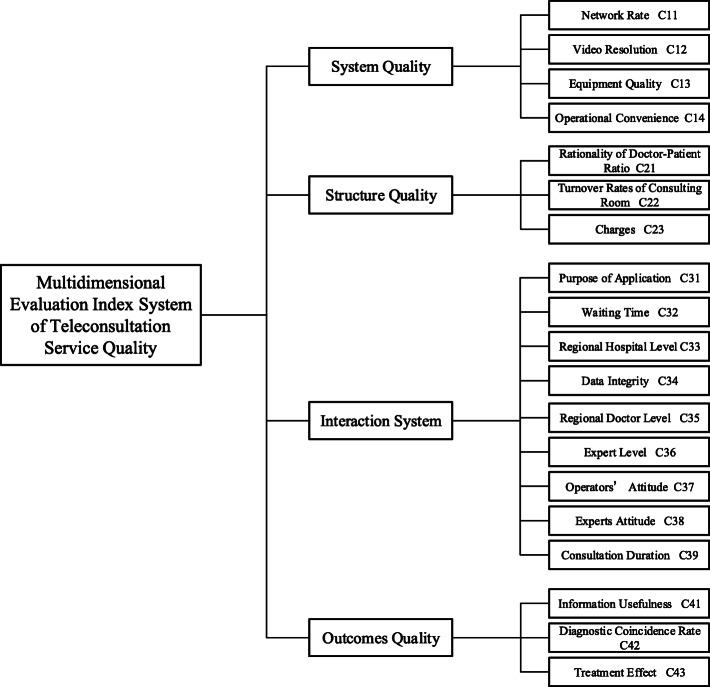
Multidimensional evaluation index system of teleconsultation service quality

In summary, the optimized multidimensional evaluation index system for teleconsultation service quality is shown in Fig. 1.

### Service quality evaluation

We conducted a survey on the quality of teleconsultation service at HTCC. The survey used anonymous methods to investigate the regional doctors’ perception and expectation of teleconsultation service. In addition, to simplify the investigation and calculation, 7- granularity linguistic evaluation term set was used in the investigation. A total of 691 questionnaires were collected and 434 questionnaires are valid (see Supplementary Files). The effective recovery rate was 62.8%.

The collected data were calculated as follows. Firstly, converting the uncertain linguistic evaluation information into trapezoidal fuzzy numbers based on Eq. (6). Secondly, calculating the perceptual and expected values of each indicator. Then obtaining the average perceptual values (AP), average expected values (AE) and their gaps according to Eqs. (10)–(12), and converting them into exact values. The results are shown in Tables 5 and 6.

**Table 5:** AP, AE, P, E and Gaps of dimensions

*C*_*ij*_	AP	AE	P	E	Gaps
*C*_*1*_	(0.607,0.684,0.761,0.838)	(0.611,0.688,0.765,0.842)	0.723	0.727	− 0.004
*C*_*2*_	(0.581,0.658,0.734,0.811)	(0.600,0.677,0.753,0.830)	0.696	0.715	−0.019
*C*_*3*_	(0.598,0.675,0.752,0.829)	(0.604,0.681,0.758,0.833)	0.714	0.719	−0.005
*C*_*4*_	(0.590,0.667,0.743,0.820)	(0.595,0.672,0.749,0.826)	0.705	0.711	−0.006

**Table 6:** AP, AE, P, E and Gaps of sub-indicators

*C*_*ij*_	AP	AE	P	E	Gaps
*C*_*11*_	(0.612,0.689,0.766,0.843)	(0.614,0.691,0.768,0.845)	0.728	0.730	−0.002
*C*_*12*_	(0.619,0.696,0.773,0.850)	(0.615,0.692,0.769,0.846)	0.735	0.731	0.004
*C*_*13*_	(0.611,0.688,0.765,0.842)	(0.610,0.687,0.763,0.840)	0.727	0.725	0.002
*C*_*14*_	(0.587,0.664,0.741,0.818)	(0.605,0.682,0.758,0.835)	0.715	0.720	−0.005
*C*_*21*_	(0.604,0.681,0.758,0.835)	(0.605,0.682,0.758,0.835)	0.720	0.720	−0.001
*C*_*22*_	(0.560,0.637,0.713,0.790)	(0.590,0.667,0.744,0.821)	0.675	0.706	−0.031
*C*_*23*_	(0.578,0.655,0.732,0.809)	(0.604,0.681,0.757,0.834)	0.694	0.719	−0.026
*C*_*31*_	(0.599,0.676,0.753,0.830)	(0.594,0.671,0.748,0.825)	0.715	0.710	0.005
*C*_*32*_	(0.609,0.686,0.763,0.840)	(0.614,0.691,0.768,0.845)	0.725	0.730	−0.005
*C*_*33*_	(0.585,0.662,0.739,0.816)	(0.581,0.658,0.734,0.811)	0.701	0.696	0.004
*C*_*34*_	(0.622,0.699,0.776,0.853)	(0.616,0.693,0.770,0.847)	0.738	0.732	0.006
*C*_*35*_	(0.593,0.670,0.747,0.824)	(0.612,0.689,0.766,0.843)	0.709	0.728	−0.019
*C*_*36*_	(0.643,0.720,0.797,0.874)	(0.631,0.708,0.785,0.862)	0.759	0.747	0.012
*C*_*37*_	(0.590,0.666,0.743,0.819)	(0.620,0.697,0.774,0.851)	0.705	0.736	−0.031
*C*_*38*_	(0.590,0.667,0.744,0.821)	(0.615,0.692,0.769,0.846)	0.706	0.731	−0.025
*C*_*39*_	(0.537,0.614,0.691,0.768)	(0.569,0.646,0.723,0.780)	0.653	0.681	−0.029
*C*_*41*_	(0.626,0.703,0.780,0.857)	(0.589,0.666,0.743,0.820)	0.742	0.705	0.037
*C*_*42*_	(0.627,0.704,0.781,0.858)	(0.591,0.668,0.745,0.822)	0.743	0.707	0.036
*C*_*43*_	(0.626,0.703,0.780,0.857)	(0.600,0.677,0.754,0.831)	0.742	0.716	0.026

**Fig. 2:**
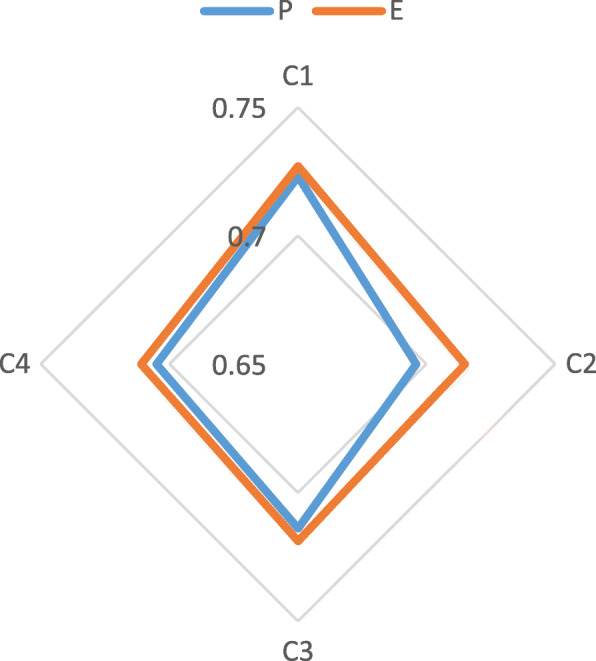
P and E of each key dimension

**Fig. 3:**
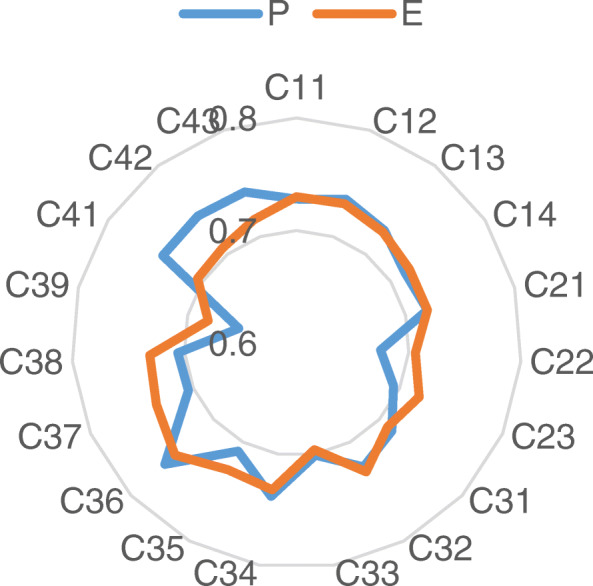
P and E of each sub-indicator

The perceptual and expected values of dimensions and sub-indicators are further drawn into a radar chart, as shown in Fig. 2 and Fig. 3. We can see that the main dimension with the highest expectation is C_1_, followed by C_3_, C_2_ and C_4_, while the highest evaluation in actual perception is C_1_, followed by C_3_, C_4_ and C_2_. It can be seen from the Fig. 3 that regional doctors have high expectation for C_36_, C_37_, C_34_, C_38_, C_12_ and C_11_, while the evaluation values of C_36_, C_42_, C_41_, C_43_, C_34_ and C_12_ are higher in the actual perception.

## Discussion

We combine Table 5, Table 6, Fig. 2 and Fig. 3 to make an in-depth analysis of the gaps and draw the following conclusions.

(1) In terms of C_1_ (System Quality), the gap between perceptual and expected value of the user is the smallest, indicating that the current teleconsultation construction has basically met the needs of system business and the users’ psychological expectation. Therefore, System Quality is an advantage project, which has a greater contribution to the development of teleconsultation, and it is necessary to maintain its advantages and strengthen its stability. Specifically, the actual perceptual values of C_12_ (Video Resolution) and C_13_ (Equipment Quality) have exceeded the users’ expectation. Although there are a few gaps between users’ perceptual values and expectation in C_11_ (Network Rate) and C_14_ (Operational Convenience), these are not the focus of attention.

(2) As for C_3_ (Interaction Quality) and C_4_ (Outcome Quality), the gaps between perceptual and expected values are small, indicating that the current construction of the teleconsultation has also basically met the users’ needs in terms of the quality of the interaction and outcome. Thus, these two aspects should be continuously improved on the premise of continuous maintenance. Among them, the actual perceptual values of C_37_ (Operators’ Attitude), C_39_ (Consultation Duration), and C_38_ (Experts’ Attitude) are quite different from the expectation, which should be paid more attention to.

(3) The gap between perceptual value and expected value in C_2_ (Structure Quality) is the largest, that is, the users’ expected value is higher, but the actual perceptual value is lower. Henan Province should focus on structure quality, optimize resource allocation in teleconsultation construction and improve the overall quality of its service. In addition, C_22_ (Turnover Rates of Consulting Room) and C_23_ (Charges) require special attention.

(4) In general, for sub-indicators, the perceptual values of C_22_, C_37_, C_39_, C_23_, C_38_ differ greatly from the expected values, that is, regional doctors think that the number of consulting rooms, attitude of the operators, consultation duration, charges and attitude of the experts during the implementation of teleconsultation are lower than their expectation. These are the key factors affecting the quality of teleconsultation service. Consequently, it is necessary to strengthen the improvement of these aspects in the subsequent construction of teleconsultation, thereby improving and promoting the service quality of teleconsultation.

According to the results of the above evaluation, it can be shown that the current construction of teleconsultation can satisfy the basic needs, but there is still room for improvement in some aspects. The results of research conducted on HTCC are widely representative, providing the following suggestions for improving the service quality in teleconsultation.

(1) Optimizing the allocation of consulting rooms according to business requirements. To expand the supply of high-quality resources and avoid wasting resources, the number of consulting rooms should be adjusted in accordance with the demands of teleconsultation to improve the turnover rate of consulting rooms, reduce waiting time, and provide timely and effective service.

(2) Improving the experience of regional doctors on the premise of ensuring the outcome quality of teleconsultation. The construction of teleconsultation needs to aim at the health of patients, optimize the quality of process and strengthen the publicity and education of relevant staff to provide better service for regional doctors and patients, which will further improve the users’ good experience, satisfaction and loyalty, and then promote the development of teleconsultation.

(3) Developing reasonable and unified charging standards and improving the charging system of teleconsultation. Costs have long been a sensitive topic. At present, there are few standardized and comprehensive charging rules in various regions, lacking a uniform standard for service price. The construction of teleconsultation should establish reasonable, unified and transparent charging standards as soon as possible according to local characteristics, and improve the charging system of teleconsultation to provide better service.

### Strengths

From the perspective of regional doctors, this paper develops a multidimensional evaluation index system for teleconsultation service quality, and optimizes the index system and evaluates service quality with the help of MGLI. Most of the existing studies have used randomized controlled experiments or exact evaluation values to evaluate the service quality in teleconsultation from the perspective of patients, without considering the perception of the real users of the teleconsultation platforms (regional doctors), the impact of mobile platform quality, and the uncertainty and preferences of decision makers. Therefore, this study is a test and beneficial supplement to previous studies. Second, medical institutions have been trying to provide better services to customers, so they need to identify shortcomings and improve them to make customers feel more satisfied. Our research helps telemedicine organizations seize the needs of customers, improve the status quo and provide better services.

### Limitations

However, there are also several limitations in this study. Firstly, the empirical data come from one region, and the sample size is limited, so further extensive empirical analysis is needed to improve the quality of teleconsultation service. Secondly, this study is a cross-sectional analysis. The concept of interviewees may change over time, so this study is just a snapshot of participants during the period of this study, and future research start with a continuous survey to explore the trends in perception of regional doctors.

## Conclusions

Based on the literature research and practical experience, an initial evaluation index system for teleconsultation service quality from the perspective of regional doctors is developed. Considering the uncertainty and ambiguity of human cognition and decision-making preferences of decision makers, MGLI is used to optimize the evaluation index system and evaluate the service quality of teleconsultation. An evaluation index system of teleconsultation service quality, including System, Structure, Interaction and Outcome, is formed. After that, this paper takes the HTCC as an example to conduct a survey on the service quality in teleconsultation based on the concept of “5GAP Model” to identify the key influencing factors. This study shows that the number of consulting rooms, attitude of operators, consultation duration, charges, and attitude of experts are the key factors affecting the quality of teleconsultation service. And we put forward suggestions for improving the quality of teleconsultation service in terms of optimizing the allocation of consulting rooms, improving regional doctors’ experience and standardizing charging standards. The research methods and results can provide important reference and guidance for improving the quality of teleconsultation service.

## Supplementary information

**Additional file 1.**

**Additional file 2.**

## Data Availability

All data generated or analyzed during this study are included in this published article and its supplementary information files.

## Abbreviations

MGLI: Multi-Granularity Linguistic Information
HTCC: Henan Province Telemedicine Center of China
